# Neural activity during working memory predicts clinical response to computerized executive function training prior to cognitive processing therapy

**DOI:** 10.1017/S0033291724003106

**Published:** 2024-12

**Authors:** Delaney Davey, Morgan M. Caudle, Samantha N. Hoffman, Amy J. Jak, Jessica Bomyea, Laura D. Crocker

**Affiliations:** 1 Department of Psychiatry, University of Illinois at Chicago, Chicago, IL, USA; 2 Research Service, VA San Diego Healthcare System, San Diego, CA, USA; 3 Joint Doctoral Program in Clinical Psychology, San Diego State University, University of California San Diego, San Diego, CA, USA; 4 Center of Excellence for Stress and Mental Health, VA San Diego Healthcare System, San Diego, CA, USA; 5 Department of Psychiatry, University of California San Diego, San Diego, CA, USA

**Keywords:** biomarkers, cognitive training, executive functioning, psychotherapy, PTSD, veterans

## Abstract

**Background:**

Executive dysfunction, including working memory deficits, is prominent in posttraumatic stress disorder (PTSD) and can impede treatment effectiveness. Intervention approaches that target executive dysfunction alongside standard PTSD treatments could boost clinical response. The current study reports secondary analyses from a randomized controlled trial testing combined PTSD treatment with a computerized training program to improve executive dysfunction. We assessed if pre-treatment neurocognitive substrates of executive functioning predicted clinical response to this novel intervention.

**Methods:**

Treatment-seeking veterans with PTSD (*N* = 60) completed a working memory task during functional magnetic resonance imaging prior to being randomized to six weeks of computerized executive function training (five 30-minute sessions each week) plus twelve 50-minute sessions of cognitive processing therapy (CEFT + CPT) or placebo training plus CPT (PT + CPT). Using linear mixed effects models, we examined the extent to which the neurocognitive substrates of executive functioning predicted PTSD treatment response.

**Results:**

Results indicated that veterans with greater activation of working memory regions (e.g. lateral prefrontal and cingulate cortex) had better PTSD symptom improvement trajectories in CEFT + CPT *v.* PT + CPT. Those with less neural activation during working memory showed similar trajectories of PTSD symptom change regardless of treatment condition.

**Conclusions:**

Greater activity of frontal regions implicated in working memory may serve as a biomarker of response to a novel treatment in veterans with PTSD. Individuals with greater regional responsiveness benefited more from treatment that targeted cognitive dysfunction than treatment that did not include active cognitive training. Clinically, findings could inform our understanding of treatment mechanisms and may contribute to better personalization of treatment.

## Introduction

Posttraumatic stress disorder (PTSD) is highly prevalent in Iraq and Afghanistan-era veterans (Fulton et al., 2015) and is associated with adverse outcomes including disability, unemployment, reduced work productivity, and poorer quality of life and physical health (Miloyan, Bulley, Bandeen-Roche, Eaton, & Gonçalves-Bradley, 2016; Schnurr, Lunney, Bovin, & Marx, 2009; Seal et al., 2011; Tanielian & Jaycox, 2008; Zivin et al., 2011). Trauma-focused cognitive behavioral therapy (CBT), including cognitive processing therapy (CPT), is one of the most effective treatments for PTSD (Bisson, Roberts, Andrew, Cooper, & Lewis, 2013; Lee et al., 2016; Watts et al., 2013). The efficacy and effectiveness of CPT has been demonstrated in a wide range of traumatized populations (Chard, 2005; Monson et al., 2006; Resick, Nishith, Weaver, Astin, & Feuer, 2002). Yet, a large portion of veterans with PTSD drop out of treatment prematurely or do not adequately respond to treatment (Bradley, Greene, Russ, Dutra, & Westen, 2005; Kehle-Forbes, Meis, Spoont, & Polusny, 2016; Schottenbauer, Glass, Arnkoff, Tendick, & Gray, 2008). One way to improve outcomes of PTSD treatment is to identify predictors of response, including biomarkers that may inform treatment mechanisms, with the ultimate goal of matching individuals to treatments where they are most likely to benefit.

A candidate predictor of PTSD treatment outcomes is executive functioning (EF) – the higher-order processes necessary for self-regulation and goal-directed behavior (Banich, 2009; Miyake et al., 2000; Snyder, Miyake, & Hankin, 2015). Intact EF skills are vital for effective CBT given the cognitive tasks involved in treatment (e.g. self-monitoring, inhibiting distorted thoughts, flexibly generating/evaluating alternative thoughts (Aupperle, Melrose, Stein, & Paulus, 2012; Crocker et al., 2018; Falconer, Allen, Felmingham, Williams, & Bryant, 2013)). Specific to PTSD, it has been posited that successful treatment involves activation of EF systems to flexibly engage, contextualize, and integrate new information or memories related to traumatic events (Foa, Steketee, & Rothbaum, 1989; Liberzon & Sripada, 2008). However, PTSD is associated with deficits in EF (Aupperle et al., 2012; Bomyea, Amir, & Lang, 2012) and corresponding frontoparietal neural circuitry (Collette, Hogge, Salmon, & Van Der Linden, 2006; Owen, McMillan, Laird, & Bullmore, 2005), including dysfunction in the amygdala, medial prefrontal cortex, hippocampus, dorsal anterior cingulate cortex (ACC), and insula (Fitzgerald, DiGangi, & Phan, 2018; Hughes & Shin, 2011; Patel, Spreng, Shin, & Girard, 2012). Cognitive elements of treatment may thus be particularly challenging for a subset of those with PTSD who demonstrated pronounced executive dysfunction. Indeed, worse baseline EF has been associated with poorer response to CBT in several disorders (D'Alcante et al., 2012; Garety et al., 1997; Granholm et al., 2008; Mohlman & Gorman, 2005), including CPT for veterans with PTSD (Crocker et al., 2018, 2023). Studies also reveal that functioning of frontoparietal substrates relevant to EF predict treatment response in trauma-focused CBT (Bryant et al., 2021; Falconer et al., 2013; van Rooij, Geuze, Kennis, Rademaker, & Vink, 2015). Taken together, this literature suggests that EF skills may impact clinical outcomes in PTSD treatment, and that EF skills could be an intervention target that is addressed prior to standard PTSD treatment in order to bolster clinical response.

A growing body of literature on computer-based cognitive training, whereby individuals complete repetitive exercises of a specific cognitive skill, demonstrates its promise for improving EF performance (Basak, Boot, Voss, & Kramer, 2008; Nouchi et al., 2013; Owen et al., 2010). While EF is an umbrella term encompassing a multitude of complex cognitive functions, the most common division of EF is comprised of three core facets, including response inhibition, updating and monitoring of working memory, and mental set shifting (Miyake et al., 2000; Miyake & Friedman, 2012). Consequently, EF training programs often incorporate exercises which target these specific EF comsponents. BrainHQ (Posit Science) is a cognitive training program available in an easily accessible online platform that shows efficacy in terms of mental health symptom reduction and/or improved cognition in a range of conditions, including attention-deficit/hyperactivity disorder (Mishra, Sagar, Joseph, Gazzaley, & Merzenich, 2016), traumatic brain injury (Mahncke et al., 2021; Voelbel, Lindsey, Mercuri, Bushnik, & Rath, 2021), unipolar and bipolar depression (Hagen et al., 2020; Lewandowski et al., 2017; Morimoto et al., 2020), substance use disorders (Bell et al., 2020; Bell, Vissicchio, & Weinstein, 2016), and mild cognitive impairment (Harvey, Zayas-Bazan, Tibiriçá, Kallestrup, & Czaja, 2022; Levy et al., 2022). Extant literature demonstrates changes in brain activation and connectivity, including frontoparietal regions, following training with BrainHQ exercises (Chen, Turnbull, Cole, Zhang, & Lin, 2022; Koshiyama et al., 2020; Lindsey et al., 2022; McEwen et al., 2023). This research supports a plasticity model of cognitive training, whereby underlying neurocircuitry changes occurring as a result of repeated practice contribute to training-related behavioral performance and clinical gains (Klingberg, 2010). Taken together, these findings collectively imply that computerized EF training via programs like BrainHQ hold promise for strengthening EF and its associated neural networks. Thus, BrainHQ EF training modules could be viable adjunctive components to PTSD intervention that may enable veterans to fully engage in and benefit from treatment.

However, addressing EF in PTSD treatment may not be a universally helpful adjunctive approach. Cognitive training for EF is mechanistically specific in that it is designed to target a particular set of thinking skills that are both deficient and etiologically involved in symptoms in a subset of individuals. Thus, EF training would likely be of greatest benefit to those with EF deficits. Consistent with this proposal, a recent meta-analysis examining the relationship between baseline cognitive abilities and cognitive training outcomes found that those with weaker baseline EF gained more from cognitive training than those who were more proficient initially (Traut, Guild, & Munakata, 2021). However, the extent to which initial neurocognitive deficits relate to interventions that combine EF training with standard psychotherapy has not been well studied to date.

In the current study, we sought to determine the extent to which the neurocognitive substrates of EF predicted clinical response to a novel cognitively enhanced CPT program. We conducted a randomized controlled trial where veterans were randomized to either computerized EF training plus CPT (CEFT + CPT) or placebo training plus CPT (PT + CPT). Prior to engaging in treatment, participants completed a baseline functional magnetic resonance imaging (fMRI) scan in which neural activity during a working memory task was assessed. Given that CEFT + CPT was a targeted intervention to ameliorate specific EF deficits prior to CPT, we expected that those with poorer EF neural activity at baseline would experience greater symptom improvement in this group. Specifically, we predicted that lower baseline neural activity in frontoparietal regions would differentially relate to clinical outcomes in CEFT + CPT *v.* PT + CPT, such that individuals with reduced baseline EF neural activity would derive greater benefit from CEFT + CPT *v.* PT + CPT.

## Methods

### Participants and procedures

The current study consisted of 60 veterans with PTSD who participated in a clinical trial comparing EF training plus CPT (CEFT + CPT; *n* = 35) to placebo training plus CPT (PT + CPT; *n* = 25; clinicaltrials.gov identifier NCT03260127) from 2018 to 2022. The sample included those who completed a 2-run working memory task while undergoing functional neuroimaging as part of the parent trial. Veteran participants were recruited primarily through VA San Diego Healthcare System (VASDHS) clinical referrals, in addition to other VASDHS research studies. Potential participants completed a phone screen, then completed a clinical assessment including written informed consent (in accordance with the VASDHS Institutional Review Board, which approved the study) to determine eligibility for the trial. The authors assert that all procedures contributing to this work comply with the ethical standards of the relevant national and institutional committees on human experimentation and with the Helsinki Declaration of 1975, as revised in 2008. Clinical raters and study therapists were blinded to condition.

Participants were included in the clinical trial if they (1) were Iraq/Afghanistan-era veterans enrolled at VASDHS, (2) were aged 18–55, (3) had a current PTSD diagnosis, (4) endorsed subjective cognitive complaints, (5) had no pending psychotropic medication changes (changes in specific medication or dosage in the previous 6 weeks or plans to change in the next month), (6) were English-speaking, and (7) had regular access to a computer to complete EF training. PTSD diagnosis was determined by administration of the Clinician-Administered PTSD Scale for DSM-5 (CAPS-5; Weathers et al., 2018) and cognitive complaints were assessed via phone screening questions that were based on the Neurobehavioral Symptom Questionnaire cognitive items (Cicerone & Kalmar, 1995). Exclusion criteria were (1) active substance use disorder in the last month, (2) suicidal intent or attempt within the last month, (3) schizophrenia, psychotic disorder, and/or bipolar disorder, (4) dementia, (5) premorbid IQ below 70, (6) participation in other concurrent PTSD intervention studies, (7) previous completion of more than four CPT sessions, (8) history of a documented neurological disorder (e.g. Parkinson's disease, multiple sclerosis, epilepsy), and (9) moderate-to-severe traumatic brain injury (TBI) according to the Veterans Affairs/Department of Defense (VA/DoD) criteria (2016). While contraindications to MRI were not exclusionary for the parent study, the current study only included those who completed the MRI scan. Of the 82 participants who were eligible, consented, and enrolled in the parent clinical trial, 64 completed the fMRI task. Four participants were excluded due to data quality issues, leaving a total of 60 participants in the current study.

Treatment randomization was stratified by mild TBI status to ensure balanced TBI history across treatment arms. Participants completed six weeks of computerized training (CEFT or PT) and then 12 sessions of standard CPT. Prior to the start of the COVID-19 pandemic in March 2020, clinical assessments and therapy sessions were completed in-person. In order to minimize risk of COVID-19 exposure, all assessments were split into two sessions during the pandemic – one completed in-person and one virtually. From March 2020 onward, all CPT sessions were completed virtually using VA telehealth software. Participants received compensation for their participation.

### Interventions

#### Computerized training

Both the active and control (‘placebo’) computerized training conditions were self-administered via BrainHQ by Posit Science (www.brainhq.com) and consisted of online training on a personal computer for 30 min a day, five times a week for six weeks. Study staff monitored training usage and progress through a secure BrainHQ portal and was in weekly contact with participants to check on their progress, answer questions, and address technical issues. The weekly check-in also served the purpose of keeping participants engaged and motivated. In addition, participants were given a $30 bonus as an incentive for completing at least 80% of the cognitive training. In the current sample, 18 participants in the PT-CPT group received the bonus and 10 in the CEFT-CPT group received the bonus. On average, participants in the PT-CPT group completed 477.35 (s.d. = 281.47) minutes, while those in the CEFT-CPT group completed 366.31(s.d. = 285.16) minutes, *t*(80) = 1.77, *p* = 0.08.

Each condition consisted of six training exercises. In the CEFT condition, the six cognitive training exercises targeted executive function processes of inhibition, shifting, and updating of working memory. The exercises were adaptive on a trial-by-trial basis based on an individual's performance. See Supplemental Materials (S1) for descriptions of each of the exercises. Participants in the PT control condition completed six exercises: crossword puzzles, sudoku, connect four, gems swap, maze races, and word search. These exercises were designed to control for nonspecific engagement and motivation of computer games; however, these games were selected to minimize the demands on executive functioning and did not provide the intensive, adaptive training of specific executive functions that the active condition provided.

#### Cognitive processing therapy

CPT is a gold-standard (trauma-focused), evidence-based treatment for PTSD (Resick et al., 2017). CPT is a 12-session protocol that involves identifying stuck points (i.e. cognitive distortions) and engaging in cognitive restructuring. Patients learn to challenge and modify stuck points related to traumatic event(s), as well as the themes of safety, trust, power and control, self-esteem, and intimacy. Participants completed CPT sessions 1–2 times a week depending on their schedule. CPT was provided by doctoral-level psychologists who completed formal VA CPT training and certification.

### Clinical outcome measure

The primary clinical outcome of interest was the PTSD Checklist for DSM-5 (PCL-5; Blevins et al., 2015), past week version, which was measured at 15 timepoints: baseline, after completion of the six weeks of computerized training (CEFT or PT), at each treatment session, and post-CPT.

### fMRI task

All participants performed an N-Back working memory task at baseline. In the task, participants monitored a continuous stream of stimuli (pseudowords) and were instructed to respond each time an item was repeated from *N* before. Each participant completed two N-back runs during fMRI, each consisting of three blocks of trials (i.e. 1-, 2-, or 3-back load conditions). Blocks were counterbalanced over runs. Pseudowords were presented in white font on a black background for 2500 ms with a 500 ms inter-item interval. The timing was constrained so that each block would last exactly 60 s. Blocks were separated by 20 s intervals (with a fixation cross; see Thomas, Duffy, Swerdlow, Light, and Brown (2022) for task details) and data were collected over two runs. The primary contrast of interest was 3-back > 1-back.

### fMRI data acquisition

Functional imaging was performed with blood-oxygen-level-dependent (BOLD) multiband whole-brain fMRI on a 3.0 Tesla GE 750 (General Electric Healthcare; Waukesha, WI) with a 32-channel head coil. Functional data were acquired using a gradient-echo echo planar imaging (EPI) sequence (TR = 800 ms, TE = 37 ms, flip angle = 52°, acquisition matrix 104 × 104, 2-mm slice thickness, no gaps, 72 ascending axial slices, multiband factor = 6, and 309 volumes per run (with the first 12 removed to achieve steady state, 2 mm isotropic voxels)). A high-resolution T1-weighted structural MPRAGE scan (TR = 8.1 ms, TE = 3.54 ms, flip angle = 8°, thickness = 0.8, no gaps, matrix = 320 mm × 320 mm, voxel size = 0.8 × 0.5 × 0.5 mm) was obtained for co-registration to the functional data and normalization.

### fMRI data preprocessing

All imaging analyses were conducted using AFNI version AFNI_22.0.15 (Cox, 1996). Preprocessing (afni.proc.py) included despiking, co-registration, spatial smoothing with a 6-mm full-width half-maximum smoothing kernel and warping to Montreal Neurologic Institute (MNI) space. Four task response regressors were generated at the individual subject level for the task reflecting trial type (1-back, 2-back, 3-back) and a high-low contrast (3-back > 1-back). The regression included censoring of large motion (≥0.3 mm) between successive time points. Prior to analysis, visual inspection for alignment and percent of censored data (<20%) was conducted, resulting in removal of four subjects who failed quality control checks. Analysis of whole-brain task effects in the sample can be found in the Supplemental Materials (S2).

### Data analysis plan

A region-of-interest (ROI) approach was used to examine the relationship between baseline neural functioning during a working memory task and change in PTSD symptoms over the course of treatment. ROIs were created using Neurosynth (Neurosynth.org; see Supplemental Materials Table S2 and Figure S2 for regions). Parameter estimates of the contrast of interest (3-back > 1-back) were extracted from each ROI and entered into separate linear mixed effects models using restricted maximum likelihood estimation procedures. Each model contained predictors of mean-centered extracted neural activity to the contrast, treatment group, time (corresponding to weeks from baseline, centered at baseline), and their respective interaction terms with subject included as a random effect. We specifically examined the extent to which neural activity at baseline was a prescriptive predictor (Fournier et al., 2009; Kraemer, Wilson, Fairburn, & Agras, 2002), that is, whether there was a significant neural activity by time by treatment condition interaction for each ROI. False discovery rate (FDR) was used to correct for multiple comparisons. In cases where a prescriptive predictor model was significant, we conducted a follow-up analysis split by treatment group to clarify the source of the effects (i.e. a neural activity × time effect separately within each group).

## Results

### Demographic, clinical, and behavioral characteristics

Table 1 presents demographic and clinical characteristics of the sample. The average age of the sample was 37.62 years (range of 23–53). Mean pre-treatment PCL-5 was 47.18 (s.d. = 14.60), indicating elevated baseline severity of PTSD. Mean post-treatment PCL-5 was 37.66 (s.d. = 20.13). Results revealed a significant decrease in PTSD symptoms over time across groups, *B* = −0.54, s.e. = .07, *p* < 0.001 (full results comparing treatment effects can be found in Crocker, 2025). Table 1 also presents average reaction time and accuracy for each trial type (1-, 2-, and 3-back). In the full sample, comparing behavioral performance across N-back trial types revealed a significant decrease in accuracy, *F*(2118) = 113.0, *p* < 0.001, and a significant increase in reaction time, *F*(2118) = 56.10, *p* < 0.001, as task difficulty increased. With regard to prior treatment for PTSD in the full sample, two participants completed minimal sessions of prior CPT (four or fewer sessions), five had completed sessions of prolonged exposure, four underwent other cognitive behavioral therapy, 11 experienced supportive therapy, and nine reported other or unknown therapies. In the PT-CPT arm, the mean length of time participants reported experiencing PTSD symptoms was 106.1 months (s.d. = 62.93), while the mean time was 137.4 months (s.d. = 91.00) for participants in the CEFT-CPT arm. There was no difference between groups in terms of time experiencing PTSD symptoms, *F*(1,58) = 2.20, *p* = 0.14.

**Table 1.** Clinical and demographic characteristics of full sample, in addition to N-back behavioral task performance variables; all values are means unless otherwise indicated and standard deviations are in parentheses

	Full sample (*N* = 60)	PT-CPT (*n* = 25)	CEFT-CPT (*n* = 35)	Statistic, *p*-value
Age	37.62 (7.81)	37.68 (7.76)	37.57 (7.95)	F(1,58) = 0.003, *p* = 0.96, *η*^2^ = <0.001
Years education	15.07 (1.99)	15.12 (2.13)	15.03 (1.92)	F(1,58) = 0.03, *p* = 0.86, *η*^2^ = 0.001
N Female (%)	13 (21.67)	3 (6.0)	10 (14.3)	Χ^2^ (2) = 3.55, *p* = 0.17, phi = 0.24
N Latino (%)	19 (31.67)	11 (22.0)	8 (11.4)	Χ^2^ (2) = 3.01, *p* = .08, phi = 0.22
Race				
White	40 (66.7)	15 (60.0)	25 (71.4)	
Black	6 (10.0)	2 (4.0)	4 (5.7)	
Asian	6 (10.0)	3 (6.0)	3 (4.3)	
Native American	5 (8.3)	2 (4.0)	3 (4.3)	
Hawaiian/Pacific Islander	3 (5.0)	1 (2.0)	2 (2.9)	
Other	8 (13.3)	4 (8.0)	4 (5.7)	
Baseline clinical symptoms				
CAPS-5	38.37 (7.78)	37.2 (8.57)	39.2 (7.17)	F(1,58) = 0.96, *p* = 0.33, *η*^2^ = 0.016
PCL-5	47.18 (14.6)	45.84 (13.68)	45.84 (13.68)	F(1,58) = 0.36, *p* = 0.55, *η*^2^ = 0.006
Post-treatment clinical symptoms				
CAPS-5	32.75 (13.95)	35.38 (11.9)	30.95 (15.24)	F(1,30) = 0.78, *p* = 0.39, *η*^2^ = 0.03
PCL-5	37.66 (20.13)	39.15 (17.26)	36.63 (22.29)	F(1,30) = 0.12, *p* = 0.73, *η*^2^ = 0.004
Number of medications	1.22 (1.87)	0.96 (1.79)	1.40 (1.92)	F(1,58) = 0.81, *p* = 0.37, *η*^2^ = 0.01
Number of mild TBIs	1.15 (1.42)	1.32 (1.58)	1.03 (1.32)	F(1,58) = 0.61, *p* = 0.44, *η*^2^ = 0.01
N-back accuracy				
1-back	0.97 (0.07)	0.96 (0.09)	0.98 (0.05)	F(1,58) = 1.01, *p* = 0.32, *η*^2^ = 0.017
2-back	0.78 (0.18)	0.76 (0.21)	0.8 (0.15)	F(1,58) = 0.93, *p* = 0.34, *η*^2^ = 0.016
3-back	0.55 (0.22)	0.58 (0.24)	0.54 (0.22)	F(1,58) = 0.45, *p* = 0.51, *η*^2^ = 0.008
N-back RT (s)				
1-back	0.64 (0.11)	0.64 (0.13)	0.64 (0.10)	F(1,58) = 0.001, *p* = 0.97, *η*^2^ = <0.001
2-back	0.95 (0.27)	0.97 (0.27)	0.93 (0.28)	F(1,58) = 0.33, *p* = 0.57, *η*^2^ = 0.01
3-back	1.05 (0.3)	0.99 (0.21)	1.09 (0.34)	F(1,58) = 1.43, *p* = 0.24, *η*^2^ = 0.02

*Note*: All clinical measures represent total scores.

PT-CPT, placebo training plus cognitive processing therapy treatment arm; CEFT-CPT, computerized executive functioning training plus cognitive processing therapy treatment arm; CAPS-5, Clinician Administered PTSD Scale for DSM-5 – Past Month; PCL-5, PTSD Checklist for DSM-5 – Past Week; TBI, traumatic brain injury.

### Relationship between neural activity at baseline and PTSD symptom change

**Figure 1.**
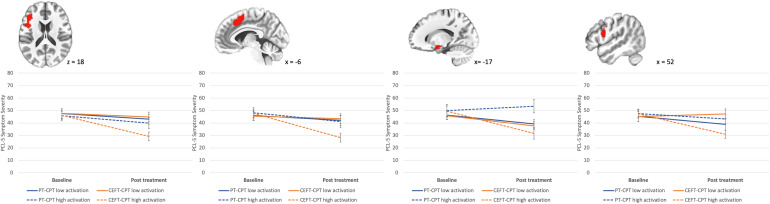
Neural activation in (a) dlPFC (L), (b) dACC, (c) amygdala (L), and (d) IFG (R) to 3 back >1 back was a prognostic predictor in treatment condition model (CEFT-CPT *v.* PT-CPT): Estimated PCL-5 scores from baseline to post-treatment sessions at 1 s.d. above and below the mean of extracted BOLD signal. ROIs reflect anatomical regions based on the Neurosynth meta-analytic mask.

Results of longitudinal models predicting PTSD symptom change from neural activity at baseline are presented in Table 2. Results of the model revealed neural activity in the left dorsolateral PFC (dlPFC), right dorsal ACC, left amygdala, and right inferior frontal gyrus (IFG). For each of these ROIs, individuals with relatively greater neural response to 3-back *v.* 1-back showed greater PTSD symptom improvement trajectories in CEFT + CPT *v.* PT + CPT, while individuals with relatively less neural response to the contrast showed similar PTSD symptom change irrespective of treatment condition (see Fig. 1). No other ROIs reached statistical significance after adjusting for multiple comparisons (*p*s > 0.02).

**Table 2.** Relationships between baseline neural activity during a working memory task and change in PTSD symptoms over treatment

	dlPFC (L)			Inferior parietal lobule (L)			Dorsal ACC (R)			Angular gyrus (R)			dlPFC (R)		
	*β*(s.e.)	*t*	*p*	*β*(s.e.)	*t*	*p*	*β*(s.e.)	*t*	*p*	*β*(s.e.)	*t*	*p*	*β*(s.e.)	*t*	*p*
ROI	−3.4 (9.11)	−0.37	0.71	−1.89 (8.29)	−0.23	0.82	4.08 (8.28)	0.49	0.62	5.30 (8.92)	0.59	0.55	1.60 (8.90)	0.18	0.86
Group	−0.18 (4.36)	−0.04	0.97	−0.003 (4.37)	1.00	<0.001	0.44 (4.35)	0.10	0.92	0.25 (4.36)	0.06	0.95	0.27 (4.49)	0.06	0.95
Time	−0.65 (0.09)	−7.42	<0.001	−0.68 (0.09)	−7.64	<0.001	−0.72 (0.09)	−8.26	<0.001	−0.72 (0.09)	−8.20	<0.001	−0.60 (0.09)	−6.73	<0.001
ROI×Time	−1.95 (0.39)	−4.98	<0.001	−1.08 (0.29)	−3.77	<0.001	−2.22 (0.34)	−6.53	<0.001	−1.55 (0.33)	−4.64	<0.001	−1.93 (0.33)	−5.76	<0.001
Group×Time	0.31 (0.14)	2.24	0.03	0.31 (0.14)	2.28	0.02	0.35 (0.14)	2.58	0.01	0.36 (0.14)	2.64	0.01	0.23 (0.14)	1.67	0.10
ROI×Group×Time	1.74 (0.57)	3.03	0.003	0.64 (0.48)	1.33	0.18	1.83 (0.64)	2.88	0.004	0.64 (0.54)	1.19	0.24	1.41 (0.60)	2.35	0.02

	Middle frontal cortex (L)			Amygdala (L)			IFG (R)			Cerebellum 1			Cerebellum 2		
	*β*(s.e.)	*t*	*p*	*β*(s.e.)	*t*	*p*	*β*(s.e.)	*t*	*p*	*β*(s.e.)	*t*	*p*	*β*(s.e.)	*t*	*p*
ROI	0.70 (6.52)	0.11	0.92	5.58 (6.72)	0.83	0.41	3.47 (6.65)	0.52	0.60	0.02 (8.28)	0.001	1.00	−5.07 (4.8)	−1.06	0.29
Group	0.23 (4.56)	0.05	0.96	0.48 (4.43)	0.11	0.91	0.10 (4.32)	0.02	0.98	0.004 (4.36)	0.002	1.00	−0.38 (4.18)	−0.09	0.93
Time	−0.064 (0.09)	−7.05	<0.001	−0.86 (0.10)	−8.53	<0.001	−0.48 (0.09)	−5.49	<0.001	−0.52 (0.09)	5.70	<0.001	−0.78 (0.09)	−9.0	<0.001
ROI×Time	−0.67 (0.23)	−2.93	0.004	−0.98 (0.29)	−3.43	0.001	−1.91 (0.23)	−8.25	<0.001	−1.79 (0.30)	6.06	<0.001	−1.13 (0.16)	−6.89	<0.001
Group×Time	0.14 (0.15)	0.88	0.38	0.75 (0.16)	4.73	<0.001	0.13 (0.14)	0.92	0.36	0.17 (0.14)	1.26	0.21	0.43 (0.13)	3.28	<0.001
ROI×Group×Timee	−0.23 (0.43)	−0.52	0.60	2.04 (0.42)	4.91	<0.001	2.11 (0.42)	5.02	<0.001	1.10 (0.57)	1.92	0.06	0.76 (0.31)	2.48	0.013

*Note*: Group represents treatment group, CEFT-CPT *v.* PT-CPT; Cerebellum 1 and 2 were two distinct regions identified within the cerebellum.

ROI, region of interest; dlPFC, dorsolateral prefrontal cortex; ACC, anterior cingulate cortex; IFG, inferior frontal gyrus.

Follow-up analyses revealed that there was a significant association between neural effects and clinical outcome in the CEFT-CPT group, but not the PT-CPT group, in the left dlPFC (CEFT-CPT: *β* = −1.99 (0.42), *p* < 0.001; PT-CPT: *β* = −0.14 (0.40), *p* = 0.72), right dorsal ACC (CEFT-CPT: *β* = −2.24 (0.36), *p* < 0.001; PT-CPT: *β* = −0.29 (0.51), *p* = .58), and right IFG (CEFT-CPT: *β* = −1.93 (0.24), *p* < 0.001; PT-CPT: *β* = −.27 (0.34), *p* = 0.44). The left amygdala ROI was associated with greater symptom reduction in both groups (CEFT-CPT: *β* = −1.03 (0.31), *p* < 0.001; PT-CPT: *β* = −0.09 (0.11), *p* < 0.001). To assist in making inferences about the directionality of neural effects relative to performance, we conducted Spearman's correlations between N-back task accuracy with the significant ROIs from our previous models. Results revealed that greater activation in the right dorsal ACC, *r* = 0.24, *p* < 0.005, and right IFG, *r* = 0.21, *p* < 0.017, was associated with better accuracy, suggesting that greater activation reflected greater working memory performance.

## Discussion

The current study sought to examine relationships between baseline neural correlates of working memory and PTSD symptom change in veterans enrolled in a randomized controlled trial testing a novel treatment combining EF training with CPT (CEFT + CPT *v.* PT + CPT). We evaluated the extent to which neural activation was a prescriptive predictor. That is, we determined if baseline neural activity differentially related to clinical PTSD outcomes measured using weekly PCL-5 scores administered throughout treatment. We found that frontoparietal regions were differentially predictive of outcomes depending on treatment group. Contrary to study hypotheses, greater (rather than less) activation of regions implicated in working memory (i.e. dlPFC, dorsal ACC, amygdala, and IFG) was associated with better PTSD symptom improvement trajectories in CEFT + CPT. In contrast, neural activity did not show statistically significant associations with PTSD symptom change in PT-CPT.

Results suggest that activity of frontal regions implicated in working memory may serve as an important biomarker of response to a cognitively enhanced treatment in veterans with PTSD. Findings from the current study are consistent with a large body of work that highlights the role of a frontoparietal network in working memory and EF (Collette et al., 2006; Owen et al., 2005). This network, in which the dlPFC and IFG are core regulatory components, facilitate ‘cold’ executive skills and processes inclusive of working memory, which regulate the series of decisions involved in the planning and execution of goal-directed behavior (Ward, 2019). The dlPFC is responsible for various working memory operations, including attention allocation and task-relevant functions such as the monitoring and manipulation of cognitive representations (Barbey, Koenigs, & Grafman, 2013; D'Esposito, Postle, & Rypma, 2000), while the right IFG is implicated in cognitive inhibition, a component of executive control defined as the suppression of inappropriate responses (Aron, Robbins, & Poldrack, 2004; Molnar-Szakacs, Iacoboni, Koski, & Mazziotta, 2005), in addition to attentional switching (Cools, Clark, Owen, & Robbins, 2002; Dove, Pollmann, Schubert, Wiggins, & von Cramon, 2000; Hampshire & Owen, 2006). Additionally, the cingulate is a key node within salience and cognitive control networks that signals the importance of external stimuli (Niendam et al., 2012). The cingulate cortex (i.e. dorsal ACC) is theorized to play an integrative role across multiple brain systems in the service of cost/benefit computations necessary for tenacity during demanding tasks (Touroutoglou, Andreano, Dickerson, & Barrett, 2020), in addition to aiding in conflict monitoring and action selection and execution (Stevens, Hurley, & Taber, 2011; Vassena, Holroyd, & Alexander, 2017; Vogt, 2019). Our correlational findings, alongside prior data examining low spans on the N-back task (Lamichhane, Westbrook, Cole, & Braver, 2020), suggest that greater activity across these regions supports working memory ability.

The present findings expand prior literature that demonstrates frontoparietal functioning relates to PTSD treatment outcomes (Bryant et al., 2021; Falconer et al., 2013; van Rooij et al., 2015). Several methodological features differ between our study and this earlier work, including treatment modality (CPT *v.* other trauma focused therapy), use of a comparator group, and task type (working memory *v.* response inhibition); these features may account for differences in findings. Results from the present study indicated that veterans who had relatively higher baseline neural response to working memory processing fared better in treatment – but this effect was specific to those receiving cognitive training in conjunction with CPT rather than placebo training paired with standard CPT. These findings align with a capitalization model of treatment response, whereby a given treatment is best matched to an individual when it builds upon their strengths (i.e. improving cognitive skill in those with greater baseline ability [Cheavens, Strunk, Lazarus, & Goldstein, 2012]) rather than attempting to target a relative weakness.

It has been posited that successful PTSD treatment involves activation of EF systems to flexibly engage, contextualize, and integrate new information or memories related to traumatic events (Foa et al., 1989; Liberzon & Sripada, 2008). For instance, CPT includes skills such as self-monitoring to identify trauma-related beliefs, inhibiting or disengaging from distorted thoughts and evaluating them, and flexibly generating alternative thoughts. These skills likely draw upon EF and the coordinated activity of regions that support these functions, including frontoparietal areas. At its core, CPT teaches clients to increase and refine the use of EF to manage emotional distress. Consequently, those with better baseline neurocognitive ability may have benefited the most from training in terms of obtaining the greatest EF gains, resulting in better downstream effects during treatment on processes linked to cognitive skills (e.g. better cognitive flexibility to modify maladaptive thoughts or inhibit negative thinking [Bomyea & Lang, 2015] and improved emotion regulation [Schmeichel, Volokhov, & Demaree, 2008]). Put differently, those with better EF brain function may capitalize on that activation during computerized EF training, priming those veterans to effectively employ EF skills in treatment and effectively reduce PTSD symptoms. Further research will be needed to clarify mechanisms by which baseline EF activation confers clinical benefit in this type of intervention.

This study has a number of limitations. First, the sample size was relatively small and consisted primarily of male Iraq and Afghanistan-era veterans. Additional research is needed in a larger and more generalizable sample to extend findings to female veterans, veterans of other eras, and to non-Veteran populations. Veterans did not complete posttreatment imaging sessions and, therefore, we can only speak to the effect of initial neural activity and cannot make inferences about the impact of treatment on working memory brain functions. Neuroimaging prediction analyses have inherent limitations, including in some cases low feasibility for utilization clinically given the high cost and participant burden. However, proxies of neural functioning (based on indirect physiological, behavioral, neuropsychological, and ecological momentary assessment measures, among others) could ultimately be helpful to identify those who would benefit most from particular treatments once neural predictors are well validated. Regarding mechanisms, we are unable to determine which aspects of the computerized executive function training translate to better treatment outcomes and, thus, future research is needed to identify the specific mechanisms by which those with greater activation is working memory-related brain regions derive benefit from cognitive training. Moreover, we utilized a working memory task, which evaluates one of three core facets of EF (Miyake et al., 2000; Miyake & Friedman, 2012); as a result, it is unclear whether similar findings would emerge for other EF tasks (e.g. inhibition).

Despite limitations, the present study is among the first to explore neural predictors of treatment-related PTSD symptom change – specifically, neural activity of a working memory task at baseline predicting clinical outcomes following a novel cognitive training intervention. Clinically, findings have the potential to inform precision medicine and increase our understanding of treatment mechanisms. Veterans with greater frontal brain activity related to working memory at baseline may be better able to utilize EF training, which could have had downstream positive impacts on psychotherapy effectiveness. Future research is needed to elucidate the mechanisms by which activity in brain regions implicated in working memory contribute to improved treatment response for those who complete computerized executive function training prior to CPT. Moreover, future studies should identify whether neural activity associated with other EF components (i.e. inhibition) also predict treatment response.

## Supporting information

Davey et al. supplementary material Davey et al. supplementary material

## Supplementary material

The supplementary material for this article can be found at https://doi.org/10.1017/S0033291724003106.
