# Supplementary material for: Neural activity during working memory predicts clinical response to computerized executive function training prior to cognitive processing therapy
Source: Psychol Med. 2024 Dec 16;54(16):4833–42. doi: 10.1017/S0033291724003106 (PMC11779553; doi:10.1017/S0033291724003106)
Supplement: Davey et al. supplementary material [file S0033291724003106sup001.docx]

**Supplemental materials for: Neural activity during working memory predicts clinical response to computerized executive function training prior to Cognitive Processing Therapy**

Delaney Davey^1,4^, Morgan M. Caudle^2^, Samantha N. Hoffman^2,4^, Amy J. Jak^3,4,5^, Jessica Bomyea*^3,4,5^, Laura D. Crocker*^3,4^

^1^Department of Psychiatry, University of Illinois at Chicago, Chicago, IL, USA

^2^Joint Doctoral Program in Clinical Psychology, San Diego State University, University of California San Diego, San Diego, CA, USA

^3^Center of Excellence for Stress and Mental Health, VA San Diego Healthcare System, San Diego, CA, USA

^4^Research Service, VA San Diego Healthcare System, San Diego, CA, USA

^5^Department of Psychiatry, University of California San Diego, San Diego, CA, USA

*Denotes co-senior authorship

**Corresponding Author:**

Jessica Bomyea

9500 Gilman Dr. MC 0855

La Jolla CA 92037

**S1. BrainHQ Module Descriptions**

1. Mind Bender: cognitive flexibility/set shifting exercise, participants switch between two different rules to make correct responses
2. Auditory Ace: auditory working memory exercise, essentially an N-back task where auditory information is retained and manipulated to determine if the current card matches a card N steps back in the sequence
3. Card shark: visuospatial working memory exercise, similar to the Auditory Ace, except matches are determined visually
4. Juggle Factory: visuospatial working memory exercise, participants reconstruct a sequence of numbers in moving circles in the correct order, at more difficult levels participants must inhibit the influence of distractors
5. Mixed Signals: inhibition exercise, participants respond if what they hear and see matches in a particular way (while ignoring irrelevant information), otherwise they withhold their response
6. Divided Attention: inhibition exercise, participants indicate whether two objects match on a particular aspect (e.g., shape, color, fill) while ignoring irrelevant/competing aspects

**S2. Whole brain analysis of task effects**

Voxel-wise percent signal change in the whole brain was entered into a t-test to evaluate regions of significant activation to the regressor of interest (3-back>1-back) as compared to baseline. Permutations testing within AFNI’s 3dttest++ (with updated ClustSim), which computes a three parameter spatial autocorrelation function from the model residuals using 3dFHWMx to create an optimal smoothing kernel, were used to guard against identifying false positive activations. A voxel-wise a priori probability of .001 with a corrected cluster-wise activation probability of .01 was considered. Results of this analysis revealed significant neural activation in regions including cingulate cortex and parietal cortex, as well as deactivation in medial prefrontal cortex (Table S1, Figure S1).

**Figure S1. Neural activation to 3back>1back contrast**


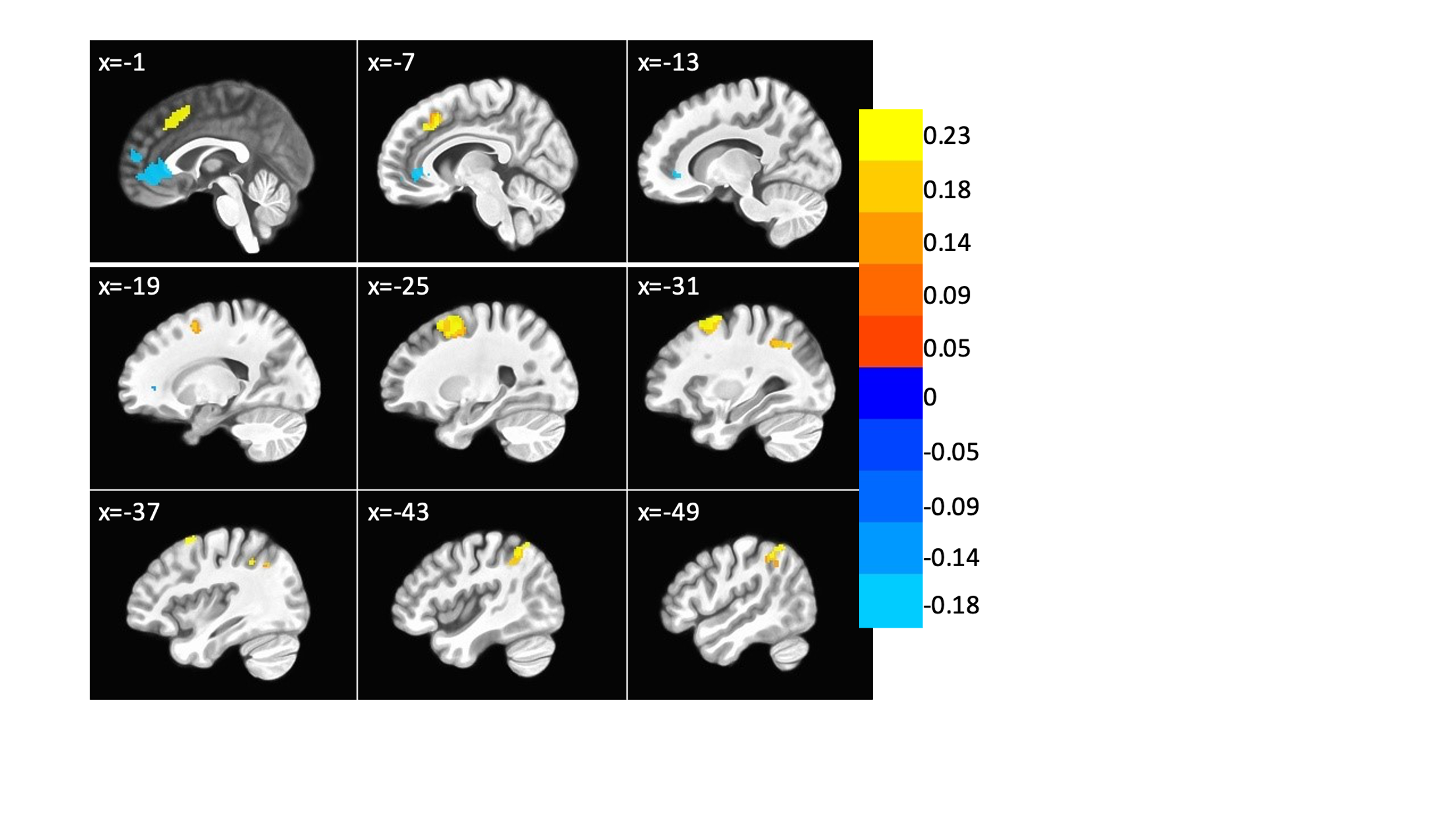
**Table S1. Regions of task-based neural activity to 3-back>1-back contrast**

| ROI Number | Volume | x | y | z | Region | BA |
| --- | --- | --- | --- | --- | --- | --- |
| 1 | 862 | -2 | 43 | -2 | vmPFC (L) | 32 |
| 2 | 514 | 0 | 22 | 26 | pre-SMA | 8 |
| 3 | 510 | 28 | 9 | 58 | Middle Frontal Cortex (R) | 6 |
| 4 | 313 | 42 | -50 | 47 | Inferior Parietal Lobule (R) | 40 |

Abbreviations: vmPFC = ventromedial prefrontal cortex; SMA = supplementary motor area

**S3. Depiction of ROI mask**

The Neurosynth association map for the term “working memory” thresholded for a false discovery rate of .01. Examination of extractions by ROI revealed extreme outliers in cerebellum attributable to signal drop out. These regions were excluded from further analysis.

**Figure S2. Regions derived from the Neurosynth working memory (Topic 045) mask**


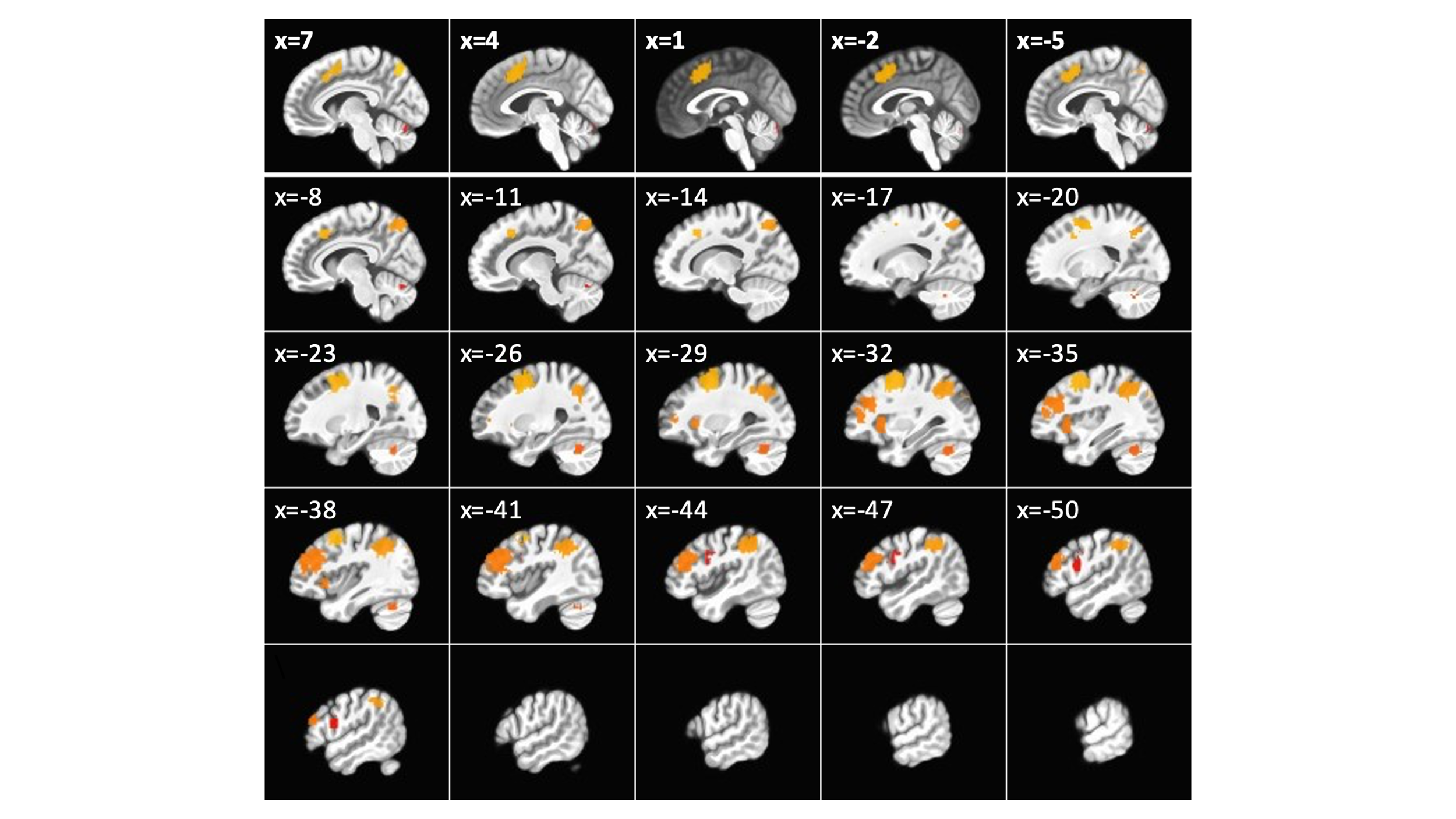


**Table S2. Regions derived from Neurosynth working memory (Topic 045) mask**

| ROI Number | Volume | x | y | z | Region | BA |
| --- | --- | --- | --- | --- | --- | --- |
| 1 | 2643 | -39 | 12 | 36 | dlPFC (L) | 9 |
| 2 | 2060 | -31 | -58 | 45 | Inferior Parietal Lobule (L) | 7 |
| 3 | 2026 | 17 | 11 | 51 | Dorsal ACC (R) | 32 |
| 4 | 1785 | 33 | -55 | 45 | Angular Gyrus (R) | 40 |
| 5 | 1675 | 40 | 35 | 20 | Insula (R) | 46 |
| 6 | 297 | 31 | -61 | -31 | Cerebellum | -- |
| 7 | 277 | -36 | 52 | 10 | Middle Frontal Cortex (L) | 10 |
| 8 | 213 | -22 | -1 | -18 | Amygdala (L) | 34 |
| 9 | 162 | 49 | 8 | 25 | IFG (R) | 44 |
| 10 | 152 | -35 | -61 | -30 | Cerebellum 1 | -- |
| 11 | 123 | -30 | 23 | 0 | Insula (L) | 13 |
| 12 | 71 | 4 | -76 | -22 | Cerebellum 2 | -- |

Abbreviations: dlPFC = dorsolateral prefrontal cortex; ACC = anterior cingulate cortex; IFG = inferior frontal gyrus
